# Bacteria Death and Osteoblast Metabolic Activity Correlated to Hydrothermally Synthesised TiO_2_ Surface Properties

**DOI:** 10.3390/molecules24071201

**Published:** 2019-03-27

**Authors:** Alka Jaggessar, Asha Mathew, Tuquabo Tesfamichael, Hongxia Wang, Cheng Yan, Prasad KDV Yarlagadda

**Affiliations:** 1 Science and Engineering Faculty, Queensland University of Technology, 2 George Street, Brisbane, QLD 4001, Australia; a.jaggessar@hdr.qut.edu.au (A.J.); t.tesfamichael@qut.edu.au (T.T.); hx.wang@qut.edu.au (H.W.); c2.yan@qut.edu.au (C.Y.); 2 Institute of Health and Biomedical Innovation, Queensland University of Technology, 60 Musk Avenue, Kelvin Grove, QLD 4059, Australia; asha.mathew@qut.edu.au

**Keywords:** hydrothermal synthesis, osteoblast response, mechanical properties, nanostructured surfaces, bactericidal surfaces, orthopaedic implants

## Abstract

Orthopaedic surgery comes with an inherent risk of bacterial infection, prolonged antibiotic therapy and revision surgery. Recent research has focused on nanostructured surfaces to improve the bactericidal and osseointegrational properties of implants. However, an understanding of the mechanical properties of bactericidal materials is lacking. In this work, the surface properties of hydrothermal TiO_2_ nanostructured surfaces are investigated for their effect on bactericidal efficiency and cellular metabolic activity of human osteoblast cells. TiO_2_ nanostructures, approximately 307 nm in height and 14 GPa stiffness, were the most effective structures against both gram-positive (*Staphylococcus aureus*) and gram-negative (*Pseudomonas aeruginosa*) bacteria. Statistical analysis significantly correlated structure height to the death of both bacteria strains. In addition, the surface contact angle and Young’s modulus were correlated to osteoblast metabolic activity. Hydrophilic surfaces with a contact angle between 35 and 50° produced the highest cellular metabolic activity rates after 24 h of incubation. The mechanical tests showed that nanostructures retain their mechanical stability and integrity over a long time-period, reaffirming the surfaces’ applicability for implants. This work provides a thorough examination of the surface, mechanical and wettability properties of multifunctional hydrothermally synthesised nanostructured materials, capable of killing bacteria whilst improving osteoblast metabolic rates, leading to improved osseointegration and antibacterial properties of orthopaedic implants.

## 1. Introduction

Titanium alloys have been widely used in the medical industry for surgical implants due to their favourable mechanical properties, corrosion resistance, passivity and biomedical properties [1,2,3,4]. In recent times, there has been a large research focus on fabricating nanostructured surfaces as a means of reducing bacterial adhesion on medical implants. Physical (micro and nanoscale topography) and chemical surface properties have been found to influence bacterial adhesion and attachment [5,6].

Factors such as the body’s acceptance of the foreign material, osseointegration and bone regrowth, influence implant integration. Surface topography is said to have a direct effect on the bone’s biological response, as micro-rough implants have shown to be superior to smooth implants [7,8]. The cells’ ability to adhere to the surface of the implant is influenced by surface properties, charge distribution and material chemistry [9]. Surface modification techniques alter the surface roughness of the implant, to mimic that of natural bone, thereby improving the implant’s integration into the body [10,11,12]. Studies have found that inducing porous structures, with morphologies similar to that of the natural extracellular matrix, benefits vascularization, improves osseointegration and bone regrowth, transports nutrients, and increases biocompatibility and implant lifespan [2,9,10,13,14,15]. Similarly, nanostructures improve cell adhesion by providing pathways for a continuous supply of the nutrients and body fluids necessary for cell growth [16].

Surface structure, morphology and wettability play key roles in the successful osseointegration of implants [7,11,17,18]. Wettability is considered one of the most important surface properties that affect the biological response to an implant [19]. It can affect bacterial adhesion and biofilm formation, protein adsorption, and the rate of osseointegration in vivo. Hydrophilic surfaces have a tendency to improve early stages of cell adhesion, proliferation, differentiation and bone mineralization, compared to hydrophobic surfaces [7,19,20,21,22,23,24,25,26]. While the antibacterial properties of TiO¬_2_ has been established, research is limited in establishing the mechanical properties of bactericidal surfaces.

In our group’s previous work, we found that nanofibers approximately 300 nm in length improved human osteoblast cell growth over 24 h compared to flat Ti-6Al-4V, and simultaneously accelerated *Staphylococcus aureus* (*S. aureus*) death over 18 h [27]. This work examines the effect of surface morphology and structure size of hydrothermally synthesised TiO_2_ surface textures on bactericidal properties and osteoblast metabolic activity. The mechanical properties, wettability and surface roughness, are statistically investigated. This study highlights the significance of nanoscale texture, topography and general surface properties in determining cellular and bacterial responses.

## 2. Results

### 2.1. Surface Morphology

**Figure 1:**
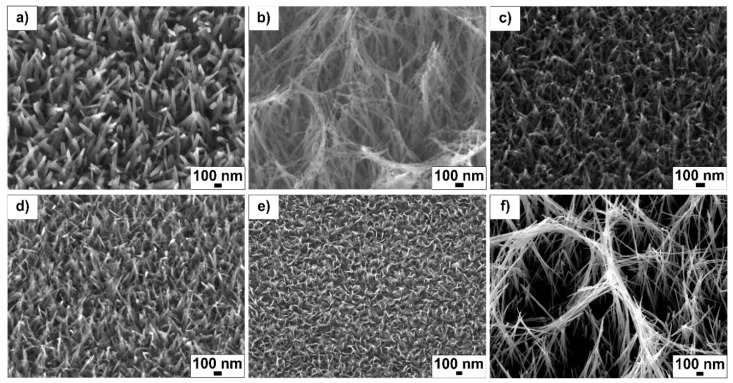
SEM images of the TiO_2_ surfaces fabricated under conditions of (**a**) 1.0_3_240, (**b**) 2.0_3_240, (**c**) 0.1_3_240, (**d**) 1.0_3_120, (**e**) 1.0_1_240 and **f**) 2.0_10_240.

Varying the NaOH concentration, hydrothermal reaction time and reaction temperature produced different TiO_2_ nanotextured surfaces. Figure 1 shows SEM topographical images of the fabricated samples. Each sample displays a unique structural distribution, spatial nanostructure arrangement and density. The array height and general morphology were heavily influenced by the process parameters, particularly NaOH concentration. Sample 1.0_3_240 (Figure 1a) produced randomly spaced and sharp-tipped individual nanowires with an average length of 307 ± 29 nm (Table 1). Sample 2.0_3_240 doubled this concentration to 2 M NaOH, producing a largely different structure (Figure 1b) compared to the 1 M NaOH reaction (Figure 1a). At this concentration, large fiber heights (1317 ± 259 nm) with an interconnected wire mesh was observed. In contrast, sample 0.1_3_240 (Figure 1c), which used low NaOH concentrations (0.1 M), produced shorter length (180 ± 40 nm) and smaller diameter (17 ± 3 nm) wires.

Sample ¬1.0_3_120 was obtained at a reaction temperature of 120 °C. The topography of this surface (Figure 1d) was similar to ¬sample 1.0_3_240 (Figure 1a); however, the tips of these nanostructures appear to have fused together. The average height of these structures was larger (328 ± 55 nm), whereas their average diameter (18 ± 3 nm) was smaller than sample 1.0_3_240. Sample 1.0_1_240 (Figure 1e) was fabricated in a short reaction time (1 h), producing a very similar shape and diameter to the 1.0_3_120 sample (Figure 1d). The structures of the 1.0_1_240 sample grew to an average height of 244 ± 36 nm and diameter of 21 ± 4 nm.

Sample 2.0_10_240 (Figure 1f) was fabricated under high concentration and temperature (2 M NaOH at 240 °C for 10 h). Similar to sample 2.0_3_240, at this NaOH concentration (2 M) the fibers grew and fused together, producing a mesh-like array. The long reaction time (10 h) was responsible for the significantly larger array dimensions compared to the same NaOH concentration and shorter reaction time (2 h, Figure 1b). All surface structures grew in a random manner in terms of the orientation angle and spacing.

The average height and diameter of the surface structures for each sample is summarised in Table 1. Three samples were fabricated for each reaction condition, with 10 height and diameter measurements taken from each sample.

**Figure 2:**
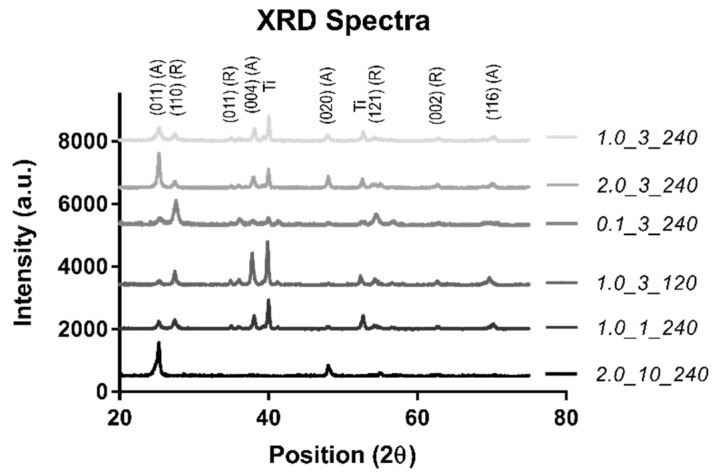
The XRD spectra.

An XRD was completed to confirm the surface material (TiO_2_) (Figure 2).

As can be seen in Figure 2, the spectra agree well with the standard rutile (R) and anatase (A) diffraction peaks, confirming that the material is TiO_2_ with a Ti substrate [28]. While all samples showed a combination of these three phases, sample 2.0_10_240 showed a majority of the anatase phase with one minor rutile peak, indicating that the phase of the nanostructured sample was predominantly anatase. The Ti peak from the substrate was not observed in sample 2.0_10_240 due to the large thickness of the TiO_2_ nanostructured surface, which obscured the substrate surface from detection.

### 2.2. Surface Roughness

Surface roughness was measured using a LEXT OLS4100 3D Laser Measuring Microscope (Olympus, Tokyo, Japan). Table 2 summarizes the average arithmetic mean deviation (S_a_) of each sample.

The results of the optical profilometry show that surface textures with small height (sample 0.1_3_240, 180 ± 40 nm) had small average mean surface roughness (0.027 µm). Similarly, sample 2.0_3_240, which had the largest average array height (14796 ± 3053 nm) had the highest average mean surface roughness (2.296 µm). For other samples, surface roughness increased with array height, with the exception of sample 1.0_3_240 (307 ± 29 nm), which had an S_a_ of 0.140 µm, similar to that of sample 2.0_3_240 (array height 1317 ± 259 nm). Overall, the results showed a large variation in surface roughness properties for different samples and morphologies. The skewness and large kurtosis values (particularly for sample 1.0_1_240) show that surface roughness is not uniform across the scanned area, indicating that the fabricated surfaces were random in nature, due to the lack of control within the hydrothermal vessel.

### 2.3. Mechanical Properties

Mechanical properties were measured using a Hysitron TI 950 Nanoindenter (Bruker, Billerica, MA, USA). The average elastic modulus and hardness values are shown in Table 3. Ti-6Al-4V (110 GPa elastic modulus and 0.36 Poisson ratio) was used to calculate the Young’s modulus of the sample material.

**Figure 3:**
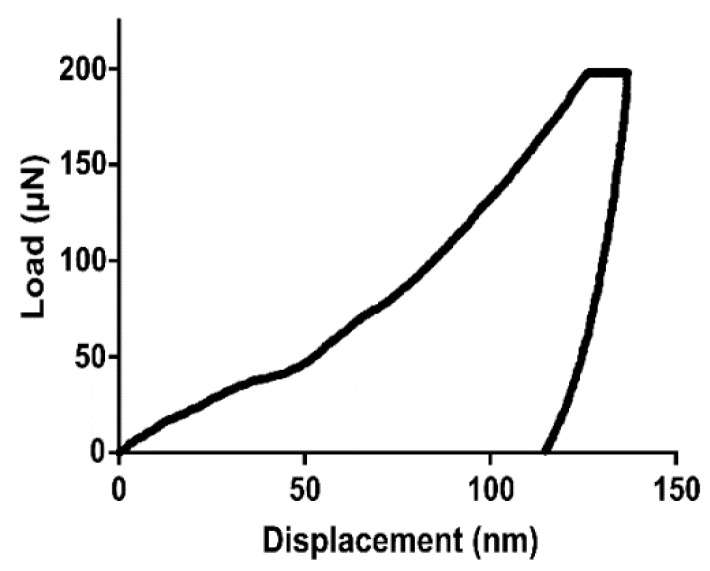
An example load vs. displacement curve obtained from nanoindentation (sample 1.0_3_120).

Surface textures with mesh-like morphologies produced structures with low Young’s moduli compared to surfaces with pillar-like structures. Sample 2.0_3_240, which had the largest array height and diameter, produced the lowest average Young’s modulus and hardness properties. Samples 1.0_1_240 and 1.0_3_120 showed the highest Young’s moduli of the sample group. Sample 0.1_3_240 produced high hardness values, potentially due to the small structure height, allowing the Ti substrate to be detected. Figure 3 shows the load vs. displacement curve obtained by the nanoindentation tests for the 1.0_3_120 sample. As in typical load vs. displacement curves, the unloading gradient (dP/dh) is indicative of the hardness properties of the surface.

### 2.4. Contact Angle

The contact angle measured the hydrophilicity of each surface. Table 4 shows the average contact angle, measured by the static sessile drop method.

Interestingly, the results showed that large mesh-like morphologies (samples 2.0_3_240 and 2.0_10_240) produced superhydrophilic surfaces (contact angle below 10°) due to the large porosity of the nanostructured surface, allowing liquid to penetrate. Structures with smaller heights (samples 1.0_3_120 and 1.0_1_240) repelled the spreading of the liquid, giving higher contact angles. In the case of sample 1.0_1_240, the contact angle was measured to be above 90°, indicating that the surface was on the borderline between hydrophilic and hydrophobic. This was the only surface out of the samples tested to produce a near hydrophobic surface.

### 2.5. Bactericidal Effects

**Figure 4:**
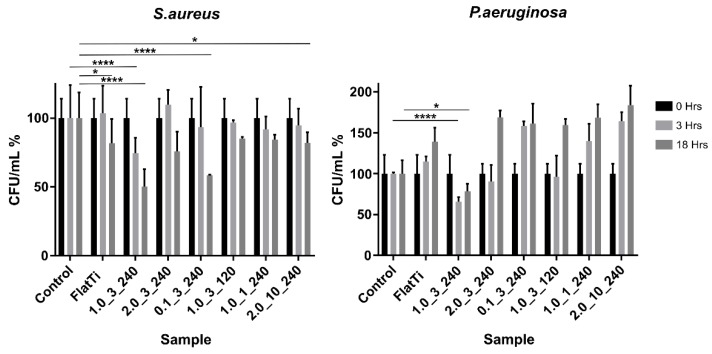
The bactericidal activity of *S. aureus* and *P. aeruginosa*, where * *p* < 0.1, ** *p* < 0.01, *** *p* < 0.001 and **** *p* < 0.0001.

The bactericidal properties of each surface against *S. aureus* and *Pseudomonas aeruginosa* (*P. aeruginosa*) were measured using the standard plate count method [29] at 0-, 3- and 18-h time points. A control well of cells alone (no substrate material) measured the natural death of the cells over the given incubation period, and a flat Ti surface was used as a comparison, representing the current implant surfaces. Figure 4 shows the bacteria viability (CFU/mL) test results of *S. aureus* and *P. aeruginosa* as a percentage of the cells alone control for each surface texture. Results that were statistically significant are indicated.

The results show that sample 1.0_3_240 was the most efficient surface, reducing the CFU/mL of *S. aureus* by 46% over 18 h. This surface significantly (*p* < 0.0001) reduced the CFU/mL compared to both the control and flat Ti-6AL-4V samples, indicating that the textured surface played a significant role in killing *S. aureus* cells. Similarly, the results showed that there was a significant difference between the CFU/mL of the flat and nanostructured surface (sample 1.0_3_240) at 3 h. This showed that the bactericidal properties of the surface had this effect on the bacteria within the 3-h time frame.

Sample 0.1_3_240 showed similar results after 18 h of incubation, with the CFU/mL dropping significantly between the 3- and 18-h time points. The results showed that the CFU/mL of this sample and the control were comparable at 3 h; however, there was a large decrease in the CFU/mL for the textured surface between 3 and 18 h. This indicated that, while samples 1.0_3_240 and 0.1_3_240 showed similar results at 18 h, the 1.0_3_240 surface produced these effects much earlier.

Sample 1.0_1_240 was the only hydrophobic surface tested (contact angle 91.12°). However, this seems to have had no significant impact on the bactericidal properties of the material, which gave similar results to sample 2.0_10_240 (superhydrophilic contact angle < 10°). Similarly, sample 2.0_3_240 (superhydrophilic mesh-like array) did not exhibit any particularly extreme results, but rather a relatively average and moderate bactericidal efficiency compared to other samples.

The sample with the largest CFU/mL of *S. aureus* at 18 h was the cells-alone control. This was an interesting finding, as it indicates that all TiO_2_ structured surfaces accelerated the death of *S. aureus* over 18 h.

The figure also shows that the CFU/mL of *P. aeruginosa* cells increased significantly over 18 h for all samples. This figure displays the CFU/mL results as a percentage of the cells-alone control sample. All *P. aeruginosa* sample wells experienced a statistically significant increase in CFU/mL from 0 to 3 h and from 3 to 18 h. Again, the sample which produced the least *P. aeruginosa* growth was 1.0_3_240, indicating that this surface texture is effective against both gram-negative and gram-positive bacteria types. This surface had significantly (*p* < 0.0001) reduced the growth of the bacteria compared to both the flat and control samples.

Unlike the observed *S. aureus* behaviour, *P. aeruginosa* cells thrived on the samples, including the cells-alone control and the flat Ti-6Al-4V. The results showed that the cells-alone control had the second lowest CFU/mL at 18 h (after sample 1.0_3_240). Sample 2.0_10_240 produced the largest CFU/mL at 18 h due to the size of the structures, which had largely increased the surface area to which bacteria could attach [5,30]. In addition, the growth rate of *P. aeruginosa* was faster than *S. aureus*, explaining the large increase in *P. aeruginosa* cells during the first 3–15 h of incubation [31,32]. The hydrophobic surface (sample 0.1_3_240) and the two superhydrophilic surfaces (samples 2.0_3_240 and 2.0_10_240) showed relatively similar behaviour, suggesting that contact angle did not significantly affect the bactericidal properties of the material.

### 2.6. Human Osteoblast Cellular Metabolic Activity

**Figure 5:**
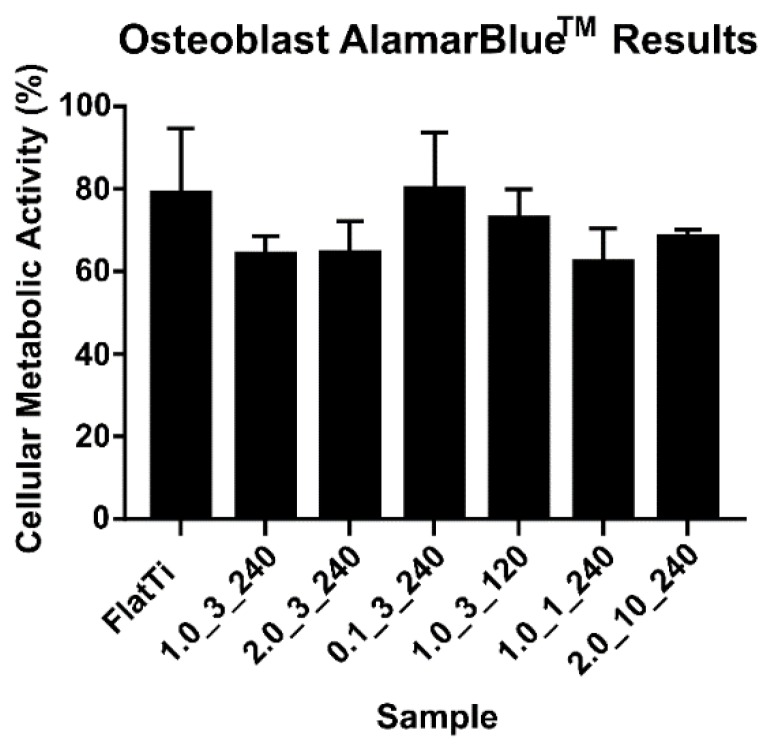
The cellular metabolic activity measured after 24 h of incubation.

The AlamarBlue^TM^ assay determined the cellular metabolic activity of human osteoblast cells on the TiO_2_ surfaces. Figure 5 shows the AlamarBlue^TM^ results after 24 h of incubation (see additional information in Supplementary file). The results have been normalised to the cells-alone control.

Figure 5 shows that flat Ti-6Al-4V and the 0.1_3_240 surface produced the highest cellular metabolic activity among the tested surfaces. Interestingly, these two surfaces have similar contact angles (50 and 36°, respectively) (Table 4), with sample 0.1_3_240 having the smallest surface structures. This indicates that the osteoblast cells had a higher cellular metabolic activity on surfaces within a specific contact angle range or a small structure height (below 200 nm).

**Figure 6:**
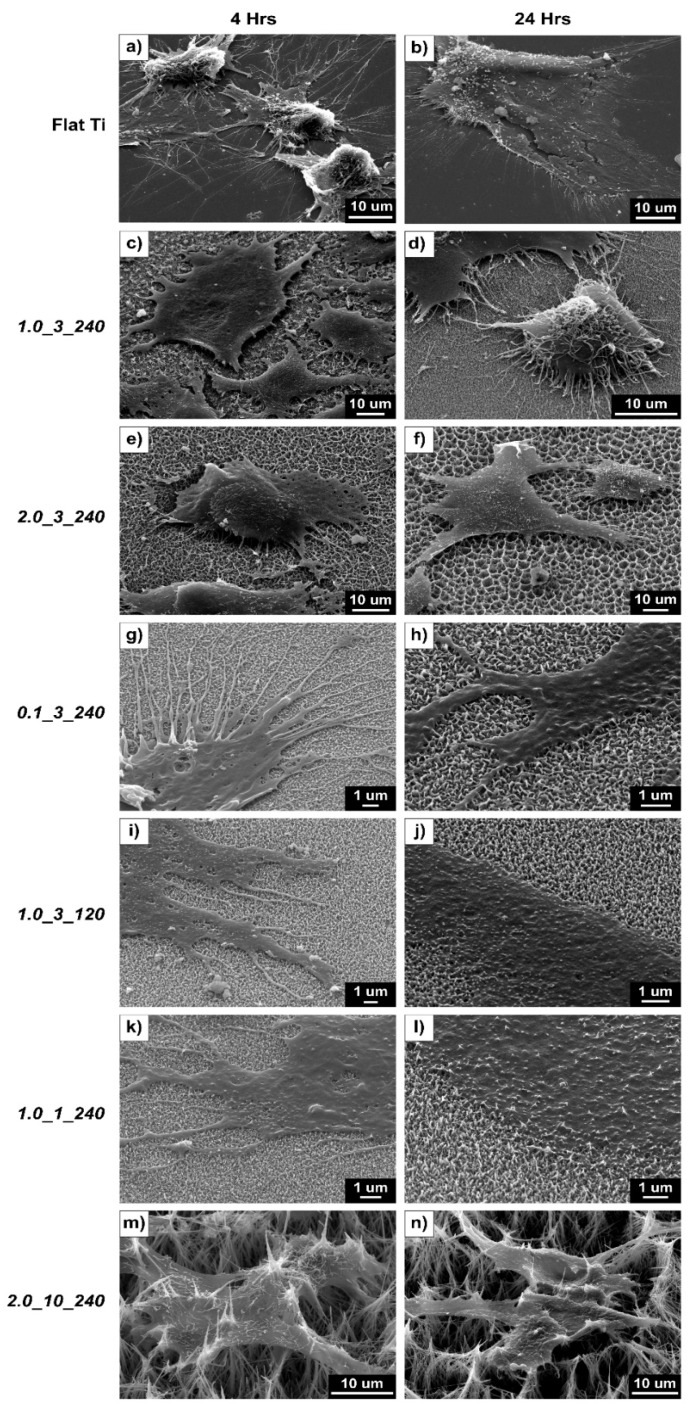
SEM images of osteoblast cells on (**a**,**b**) flat Ti, (**c**,**d**) 1.0_3_240, (**e**,**f**) 2.0_3_240, (**g**,**h**) 0.1_3_240, (**i**,**j**) 1.0_3_120, (**k**,**l**) 1.0_1_240 and (**m**,**n**) 2.0_10_240: The left-hand column contains images of the cells after a 4-h incubation, and the right column presents images of the cells after a 24-h incubation.

Figure 6 shows the SEM images of osteoblast cells attached to the flat and nanostructured surfaces after 4 and 24 h of incubation. The figure shows the effective spreading and growth of the cells on flat surfaces, which is reflected in the metabolic activity results (Figure 5). Images for samples 1.0_1_240 and 2.0_10_240 show that structures pierce the osteoblast cells, explaining the low activity rate for these samples. Alternatively, sample 0.1_3_240 showed less piercing (due to the small structure size and surface roughness of the sample), giving the highest metabolic activity of the textured surfaces.

**Figure 7:**
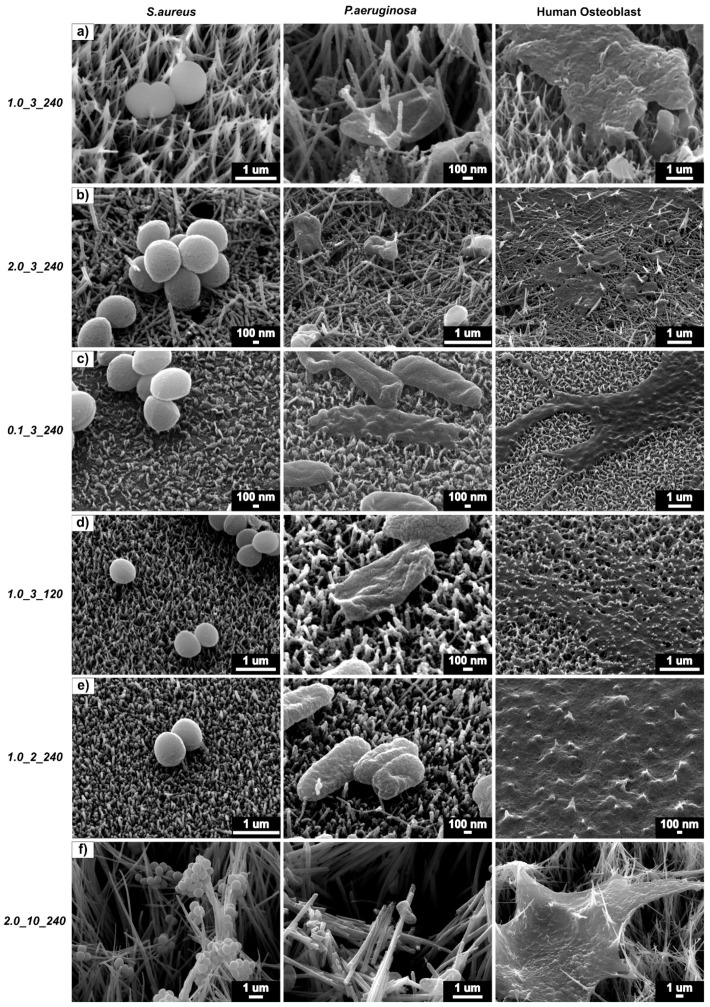
*S. aureus* (left), *P. aeruginosa* (middle) and 24 h osteoblast (right) cells on (**a**) 1.0_3_240, (**b**) 2.0_3_240, (**c**) 0.1_3_240, (**d**) 1.0_3_120, (**e**) 1.0_1_240 and (**f**) 2.0_10_240.

Figure 7 shows a direct comparison between each surface and their contact with *S. aureus*, *P. aeruginosa* and osteoblast cells. It is interesting to note that the surface 1.0_3_240 produced the best results against both bacteria types and a modest behaviour in promoting osteoblast activity. Figure 7a shows the two bacteria cells types being pierced by the nanostructures, whereas the osteoblast cell did not experience this. Sample 2.0_3_240 (Figure 7b), which has a high CFU/mL of both *S. aureus* and *P. aeruginosa* and a low cellular activity, showed both bacteria and osteoblast cells being pierced by the structures. Figure 7f shows that the large surface area of the structures provides more surfaces to which the bacteria can adhere, showing that bacteria adhere on all locations of the structures and stretch and pierce the osteoblast cells. Shorter structure heights cause less damage to the *S. aureus* cells (Figure 7c–e) but effectively puncture *P. aeruginosa* and osteoblast cells. From this figure and the results obtained from the bacteria viability and AlamarBlue^TM^ tests, it can be said that sample 1.0_3_240 was the most effective surface in killing gram-negative and gram-positive bacteria and promoting osteoblast activity.

### 2.7. Statistical Correlations

Statistical analysis completed in the IBM SPSS Statistics program tested correlations between CFU/mL (*S. aureus* and *P. aeruginosa*) and cellular metabolic activity, and the measured the surface properties. As these relationships were not linear, nonparametric correlation tests were used. Table 5 shows the correlation coefficients between the cellular metabolic activity and CFU/mL and each surface property, where 0.1 < |r| < 0.3 indicates a small or weak correlation, 0.3 < |r| < 0.5 shows a medium or moderate correlation, and 0.5 < |r| is a strong or large correlation.

The results of this correlation test showed that both the contact angle and Young’s modulus had a statistically significant (*p* < 0.01) moderate correlation to osteoblast cellular metabolic activity. This is a significant finding as it shows that the cellular metabolic activity of osteoblast cells is statistically influenced by surface properties. The correlation data shows that the metabolic activity is more strongly correlated to Young’s modulus compared to the surface contact angle. Interestingly, these results show that the mean surface roughness is not correlated to the metabolic activity of the osteoblast cells over 24 h.

The correlation tests also showed that both the CFU/mL of the gram-negative and gram-positive bacteria tested was statistically (and significantly) correlated to the height of the surface structures. The correlation was statistically weak for *S. aureus* and moderate for *P. aeruginosa*; however, both correlations are significant (*p* < 0.05). There are no other tested surface properties that are significantly correlated to the CFU/mL of these bacteria. This is an important finding, as it has the potential to influence future surface texture design.

These findings are significant as it statistically confirms the phenomenon of surface morphology and wettability playing key roles in implant osseointegration [7,11,17,18]. It has been previously believed that surface roughness influences the death of bacteria on nanostructured surfaces; however, these results show that the structure height influences bacteria death.

## 3. Discussion

The purpose of this study was to test the effect of NaOH concentration, reaction time and reaction temperature on the surface fabrication of hydrothermally synthesised TiO_2_ structured surfaces. In addition, the bactericidal properties of these surfaces to gram-negative and gram-positive bacteria and their human osteoblast metabolic activity were tested. Changing the hydrothermal process parameters produced various surface textures. The tests showed that the structure height grew with hydrothermal reaction time [31,32]. The reaction temperature had a modest effect on the structure height, with higher temperatures producing smaller structures and with larger diameters than surfaces formed at low temperatures (120 °C).

From the data collected, it can be said that NaOH concentration had the largest effect on the array height within the range of concentrations tested (0.1–2 M NaOH). At low concentrations (0.1 M), structures were less than 200 nm in height and 18 nm in diameter. These small structures also produced the lowest surface roughness (0.027 µm), with a contact angle of 41.4° and the highest osteoblast cellular metabolic activity after 24 h of incubation.

When the NaOH concentration was increased to 2 M NaOH, a highly dense, closely packed mesh-like array formed [33], due to the increased number of nucleation sites. The most significant increase in array height occurred when a high NaOH concentration (2 M) was combined with a long reaction time (10 h) and a high reaction temperature (240 °C). Surfaces with mesh-like morphologies (samples 2.0_3_240 and 2.0_10_240) produced large dimensions (height and diameter) and air pockets, resulting in high surface roughness characteristics and superhydrophilic contact angles. The large surface structures of these samples allowed liquid to penetrate the roughness grooves, reducing the wettability angles [20]. Morphological and structural parameters have a significant effect on the wetting angles, where adjusting the scale or roughness of the topography can largely impact the wetting ability [34]. This is reflected in the results, where various surface roughness values and textures produced surfaces with varying contact angles and wetting characteristics.

All surfaces contained anatase and rutile phases, indicating TiO_2_. The largest sample (sample 2.0_10_240) gave an XRD spectrum of predominately anatase. While it can be thought of as the less stable TiO_2_ phase, anatase is quite often produced during TiO_2_ formation due to its constrained structure and enhanced formation kinetics [35].

The nanostructured TiO_2_ surface caused the cells to respond to the physical morphology and interaction forces of the structures [29], leading to an effective bactericidal activity and an increased osteoblast activity compared to flat Ti, as seen in the experimental results. The deformation of the peptidoglycan cell walls by the TiO_2_ nanotextured surfaces resulted in cell death [36]. From the SEM images obtained of the bacteria cells in contact with the nanostructure surfaces, piercing of the cell walls was clearly observed. The piercing led to the disfiguration and collapse of the bacteria cells, resulting in bacteria death and highlighting this as the possible mechanism of bacteria killing. The reason for this behaviour can be explained by the stiffness of the bacteria cells compared to the TiO_2_ nanostructures. *S. aureus* and *P. aeruginosa* have a bacteria cell stiffness between 10–100 MPa [37], whereas the stiffness of the 1.0_3_240 structures was 14.4 GPa. This large difference in stiffness caused the cells to be easily pierced by the TiO_2_ nanostructures, causing cell death. The high killing efficiency of the nanostructured surface against *S. aureus* appears practical in inhibiting initial bacteria adhesion towards gram-positive bacteria, preventing further bacterial infections on implant surfaces. This is an important finding as the killing of gram-positive bacteria cells has proven difficult [38,39]. At this stage, it is unclear whether the physical size of the bacteria cell has an impact on the bactericidal efficiency of the nanostructured surface. Studies currently postulate that the difference in the bactericidal efficiency of nanostructured surfaces against gram-negative and gram-positive microbes is due to the higher number of peptidoglycan layers in the cell wall of gram-positive bacteria [36]. This results in a higher wall stiffness of the gram-positive cell, making it more difficult to kill than its gram-negative counterparts. To improve the accuracy of the plate-count method, sonication of the sample after incubation could be added to reduce the error in CFU/mL by eliminating non-adhered bacteria [40,41]. In addition, *P. aeruginosa* cells reached a stable growth rate at approximately 14 h of incubation [31], during which the rod-shaped cells divided, rapidly increasing the bacteria concentration in the suspension. Round *S. aureus* cells took longer to approach this stable growth (approximately 15–16 h) [32], resulting in the comparatively high growth of *P. aeruginosa* colonies observed in these tests.

Sample 1.0_3_240 was the most effective surface against both *S. aureus* and *P. aeruginosa*. It appears that this structure height (307 nm) and morphology was effective at piercing and stretching bacteria cells, resulting in cell-wall rupture and death [36]. This phenomenon was not observed with osteoblast cells, which remained intact. It has previously been reported that surface roughness has a large impact on the bacterial attachment of *S. aureus* and *P. aeruginosa* on titanium thin films [39]. This study found that structure height was also significantly correlated to the bacteria death of these two pathogens. 

Sample 1.0_3_240 produced higher cellular metabolic activity rates than the flat surfaces at 4 and 24 h. As the surface of medical implants is currently flat Ti-6Al-4V, the results indicate that osteoblast cell activity may increase with the presence of nanostructured surfaces. This enhancement of osteoblast cell growth by the nanostructured surfaces may be due to the increased surface area which improves cell migration and attachment as compared to flat surfaces [42].

The osteoblast cellular metabolic activity was not significantly affected by the array height or surface roughness. Statistical analysis showed that surface wettability played a significant role in determining the cellular metabolic activity after 24 h. The highest metabolic activity was observed on surfaces hydrophilic in nature with a contact angle between 35–50°, supported by previous studies [20,43]. This finding is significant as most clinical implants are currently hydrophobic in nature [7,44] and can assist future implant design in improving osteoblast cell activity. This could lead to reductions in orthopaedic surgery recovery time, improving osseointegration, bone remodeling and implant fixation [45].

## 4. Materials and Methods

This section outlines the methodology used to fabricate, characterize and test surface textures for bactericidal properties and osteoblast cellular metabolic activity. As titanium and its alloys are commonly used as implant material [3,46], flat polished Ti-6Al-4V was chosen as the control surface. Hydrothermal treatment with a NaOH reaction precursor was chosen as the fabrication method, for its reliable, efficient and environmentally friendly nature [4,47,48]. The NaOH concentration (0.1–2.0 M), hydrothermal reaction time (1–10 h) and hydrothermal reaction temperature (120–240 °C) were varied to produce different nanostructured surfaces. These surfaces were characterized using scanning electron microscopy (SEM), X-Ray diffraction (XRD), optical profilometry and wettability (surface contact angle with water), investigating the effect of the fabrication conditions on these properties. A standard plate-count method was used to test the bacterial viability of gram-positive *S. aureus* and gram-negative *P. aeruginosa* after 3 and 18 h. Lastly, AlamarBlue^TM^ assays determined the cellular metabolic activity of human osteoblast cells of each surface after 24 h, finding the most effective surface for promoting osteoblast activity.

### 4.1. Nanotextured Surface Production

Titanium plates 1 cm^2^ in size were polished to a 0.04 µm surface roughness and sonicated in acetone for 10 minutes. The samples were rinsed thoroughly with 18.2 MΩ H_2_O and dried using N_2_ gas. The plates were then placed on an angle in a custom-made polytetrafluoroethylene (PTFE) holder and reacted with 60 mL NaOH in a 125 mL Parr acid digestion vessel at a given temperature (the experimental conditions are listed in Table 6). The vessel was removed after the specified reaction time and left to cool to room temperature. Once cooled, the samples were removed, rinsed with 18.2 MΩ H_2_O and annealed for 1 h at 300 °C in a furnace. The samples were then submerged in 0.6 M HCl for 30 minutes, rinsed three times in H_2_O and finally calcined for 2 h at 600 °C. Table 6 shows the hydrothermal experimental conditions used.

### 4.2. Surface Characterisation

#### 4.2.1. Physical Properties

A JSM-7001F SEM (JEOL, Tokyo, Japan) was used to image the surface morphology of each sample. The images were taken at a 40° tilt angle, with a 15-kV accelerated voltage. The height and diameter measurements were taken using inbuilt measurement functions on the JEOL FE-SEM PC-SEM/EOS firmware VSM0055-22 (JEOL, Tokyo, Japan). A minimum of 10 measurements (for each height and diameter) were taken at random from 3 different surfaces for each experimental condition.

The contact angle was measured to determine the hydrophilicity characteristics of each surface and was used to investigate the role of the contact angle on the relationship between bactericidal efficiency and osteoblast cellular metabolic activity. The contact angle was measured using an FTA 200 Contact Angle Instrument with FTA32 software version 2.1 (First Ten Angstroms, Portsmouth, VA, USA) by the static sessile drop method (a well-established and widely used method for testing the contact angle of a surface). Water droplets were pumped out of the syringe at a rate of 2.00 µL/s until a single drop left the tip of the syringe. The angle between the water droplet and the surface was measured 2 seconds after surface contact. Both the right and left angles were measured for each droplet, with 3 measurements taken for each sample. The samples were rinsed with ethanol and dried between measurements.

Optical profilometry was used to measure the surface roughness characteristics of all fabricated surfaces. It investigated the effect of the hydrothermal process conditions on surface roughness and on bacteria and osteoblast response. The average surface roughness (S_a_) was evaluated using a LEXT OLS4100 3D Laser Measuring Microscope (Olympus, Tokyo, Japan). The samples were observed under a 100× microscopic lens, and the surface roughness measured with the Olympus LEXT OLS4100 Application 3.1.9 (Olympus, Tokyo, Japan). Optical profilometry was selected for obtaining this data as a noncontact form of characterization, eliminating surface damage.

A Rigaku SmartLab diffractometer (Rigaku, Tokyo, Japan) was used to complete XRD characterization. A Cu source, operating at 40 kV and 40 mA was run in parallel beam mode in conjunction with a Hypix 3000 detector (Rigaku, Tokyo, Japan) (0D mode). An incident angle of 2° and a scan axis of 2ϴ were selected. The patterns were collected for 1–24 h (depending on the film thickness) between a 5–75° 2ϴ range [49,50,51].

#### 4.2.2. Mechanical Properties

The Hysitron TI 950 Nanoindenter (Billerica, MA, USA) was used to measure the Young’s modulus and hardness of each sample. A Berkovich tip indented 10 different points of the sample. This tip is a three-sided pyramidal tip (100 nm tip radius and 142.3° included angle [52]). Fused silica was used to calibrate the tip area function. A trapezoid load function (loading rate of 20 μN/s and peak load of 200 μN) was applied at each measurement point. The loading rate was applied over 10 s, with the peak load held for 5 s before unloading.

The hardness for each sample was calculated using Equation (1) [52,53].

*H* = *P_max_*/*A_c_* (1)

where H is the hardness of the material, *P_max_* is the maximum applied load and *A_c_* is the contact area. 

The Young’s modulus was calculated using Equation (2) [52,53]:

1/E_r_ = (1 − v_m_^2^)/E_m_ + (1 − v_i_^2^)/E_i_ (2)

where *E_r_* is the reduced modulus measured during the test, *E_m_* is the elastic modulus of the material being measured and *v_m_* is the Poisson ratio of the material. *E_i_* and *v_i_* are the elastic modulus and Poisson ratio of the indenter tip, respectively. The *E_i_* and *v_i_* of the standard Bruker Berkovich diamond indenter tip used was 1140 GPa and 0.07, respectively [53]. The *v_m_* of the TiO_2_ structures were assumed to be equal to the Poisson’s Ratio of bulk TiO_2_ (0.28) [52].

### 4.3. Bacterial Viability Testing

Gram-positive *S. aureus* (ATCC 25923) and gram-negative *P. aeruginosa* (ATCC 27853) were selected to test the bacterial viability against the various surface textures. A standard plate count method was used to test the bacterial viability, as used in our previous work [27,29].

The samples were fabricated according to the conditions in Table 6 and sterilized in ethanol overnight and then under UV light for 20 minutes. The bacteria cells were suspended in 5 mL of sterile nutrient broth, growing to an OD_600_ of 0.3. The OD_600_ was then diluted to 0.1 using sterile phosphate buffered saline (PBS). These re-suspended cells were further diluted to 1:10 and 1 mL incubated with each sample in triplicate at 37 °C. At certain time points (0, 3 and 18 h), each suspension was sampled and appropriately diluted, and 100 µL were spread on nutrient agar plates. These plates were then incubated at 37 °C for another 24 h. The colonies were counted using ImageJ version 1.8.0_112 software (National Institutes of Health, Bethesda, MD, USA), and the colony forming units (CFU)/mL were calculated. After testing, the remaining bacteria were fixed onto the surface using 3% glutaraldehyde and washed twice with PBS. The cells were dehydrated using 1% osmium and an ethanol series (50, 70, 90 and 100%). Hexamethyldisilazane (C_6_H_19_NSi_2_) was then added to the sample wells and left to evaporate. The samples were mounted onto aluminium stubs, gold sputter-coated (using a Leica EM SCD005 (Leica, Wetzlar, Germany) sputter coater for 75 seconds) and then imaged under SEM. Each test was repeated for each bacteria strain to confirm results. A statistical analysis was completed with a two-way ANOVA, Tukey’s multiple comparison test using GraphPad Prism 7 software (GraphPad Software, San Diego, CA, USA). *P*-values of <0.05 were considered significant.

### 4.4. Osteoblast Cellular Metabolic Activity

#### 4.4.1. Cell Culture and AlamarBlue^TM^ Assay

Female human knee osteoblasts were collected after ethics approval (QUT Human Research Ethics Committee approval number 1400001024). Non-osteoarthritic bone was minced and rinsed with PBS, supplemented with Penicillin-Streptomycin (Pen/Strep) and vortexed until the PBS appeared clear after shaking. The bone chips were transferred to a tissue culture flask with culture medium and left to incubate at 37 °C until osteoblast outgrowth was observed. The cells were expanded, with the 6th and 7th passages used for testing (P6 used for initial test and P7 used in the repeated test). The culture medium was composed on MEM-alpha supplemented with Pen/Strep and 10% fetal bovine serum (FBS).

Cellular metabolic activity was tested and calculated using the AlamarBlue^TM^ assay, where a reduction in dye fluorescence was treated as proportional to cellular metabolic activity of the osteoblasts [54]. The cells were seeded onto each surface texture (10 000 cells/well) in a 24-well plate. Each well contained 500 µL of cell culture medium and was incubated at 37 °C for 4- and 24-h time periods. A well of cells only (no substrate surface) was used as the experimental control. The AlamarBlue^TM^ assay was completed in accordance with the manufacturer’s directions. At each time point, the culture medium was removed from each well, washed three times with sterile PBS, mixed with 10% AlamarBlue^TM^ solution and incubated at 37 °C for 2 h in a 96-well plate. The absorbance at 500 and 595 nm was then read using a Bio-Rad Benchmark Plus^TM^ microplate reader (Bio-Rad, Hercules, CA, USA). The percentage of dye reduction was calculated, where the metabolic activity of each flat and fabricated surface texture was normalised to the cells-alone control. The cells were then fixed onto the surfaces using the method described in the previous section. The AlamarBlue^TM^ assays were repeated on each surface structure to confirm the results. A statistical analysis was completed with a two-way ANOVA, Tukey’s multiple comparison test using GraphPad Prism 7 software (GraphPad Software, San Diego, CA, USA), with *P* values of <0.05 considered significant.

#### 4.4.2. Statistical Correlations

IBM SPSS Statistical Software version 25 (IBM, Armonk, NY, USA) was used to test the statistical correlations between the cellular metabolic activity and CFU/mL (of *S. aureus* and *P. aeruginosa*) and the previously mentioned surface properties. Since the data obtained were largely nonparametric, Spearman’s rho correlations were used.

## 5. Conclusions

Samples of different surface morphology, roughness, wettability and mechanical properties were produced using hydrothermal synthesis. The samples with small height and diameter, and a contact angle between 35 and 50° improved the cellular metabolic activity of human osteoblast cells after 24 h. It was found that the contact angle had a statistically significant correlation to the metabolic activity of the osteoblast cells. Furthermore, structure height was correlated to the death of *S. aureus* and *P. aeruginosa* bacterial cells after 18 h of incubation. The overall results provided an overview of the ways in which fabrication conditions influenced the properties of hydrothermally synthesised surface textures and, therefore, bactericidal efficiency and osteoblast metabolic activity.

## Figures and Tables

**Table 1:** A summary of the nanostructure dimensions.

Sample	Height (nm)	Diameter (nm)
1.0_3_240	306.53 ± 29.06	41.48 ± 9.23
2.0_3_240	1316.70 ± 258.95	33.40 ± 5.71
0.1_3_240	180.37 ± 40.15	17.27 ± 2.74
1.0_3_120	328.37 ± 54.53	18.35 ± 3.17
1.0_1_240	244.20 ± 36.42	20.83 ± 3.86
2.0_10_240	14796 ± 3053.26	154.07 ± 32.26

**Table 2:** The optical profilometry measurements for the surface roughness of the fabricated surfaces.

Sample	Arithmetic Mean Deviation (S_a_) (µm)	Skewness of the Roughness Profile (S_sk_)	Kurtosis of the Roughness Profile (S_ku_)
1.0_3_240	0.040	0.266	3.936
2.0_3_240	0.140	0.035	3.694
0.1_3_240	0.149	−0.743	9.192
1.0_3_120	0.027	0.362	10.534
1.0_1_240	0.051	2.653	37.366
2.0_10_240	2.296	0.362	3.161

**Table 3:** Young’s modulus and hardness measurements by nanoindentation.

Sample	Average Young’s Modulus E_m_ (GPa)	Average Hardness (MPa)
1.0_3_240	14.35 ± 2.53	14.76 ± 1.78
2.0_3_240	1.10 ± 0.22	18.32 ± 3.86
0.1_3_240	10.20 ± 1.06	1015.08 ± 160.72
1.0_3_120	32.03 ± 6.74	221.34 ± 21.04
1.0_1_240	32.04 ± 5.80	45.50 ± 3.31
2.0_10_240	0.21 ± 0.06	0.50 ± 0.12

**Table 4:** The average contact angle for each surface texture.

Sample	Contact Angle (°)	Hydrophilicity
Flat Ti	49.56 ± 6.37	Hydrophilic
1.0_3_240	14.31 ± 0.99	Hydrophilic
2.0_3_240	<10 (8.93 ± 1.19)	Superhydrophilic
0.1_3_240	35.75 ± 7.67	Hydrophilic
1.0_3_120	74.84 ± 11.38	Hydrophilic
1.0_1_240	91.12 ± 3.17	Hydrophobic
2.0_10_240	<10 (6.10 ± 1.43)	Superhydrophilic

**Table 5:** The correlation coefficients, where * *p* < 0.1 and ** *p* < 0.01 indicate a statistical significance.

	Correlation Coefficient (r)					
Characteristic	Structure Height (nm)	Structure Diameter (nm)	Young’s Modulus (GPa)	Hardness (MPa)	Contact Angle (°)	Average Surface Roughness (µm)
**Cellular Metabolic Activity**	0.113	−0.017	−0.485 **	0.098	−0.398 **	0.049
***S. aureus*** **CFU/mL (18 h)**	−0.129 *	−0.057	−0.102	0.016	−0.157	−0.076
***P. aeruginosa*** **CFU/mL (18 h)**	0.332 *	−0.053	−0.153	0.121	0.018	0.021

**Table 6:** The hydrothermal experimental conditions.

Sample Name (Conc (M)_Time (h)_Temp (°C))	NaOH Concentration (M)	Reaction Time (h)	Reaction Temperature (°C)
1.0_3_240	1	3	240
2.0_3_240	2	3	240
0.1_3_240	0.1	3	240
1.0_3_120	1	3	120
1.0_1_240	1	1	240
2.0_10_240	2	10	240

## Supplementary Materials

The following are available online at https://www.mdpi.com/1420-3049/24/7/1201/s1, Table S1: *S. aureus* average CFU/mL results; Table S2: *P. aeruginosa* average CFU/mL results; Table S3: AlamarBlueTM test results averaged.
