# Supplementary material for: Bacteria Death and Osteoblast Metabolic Activity Correlated to Hydrothermally Synthesised TiO2 Surface Properties
Source: Molecules. 2019 Mar 27;24(7):1201. doi: 10.3390/molecules24071201 (PMC6480334; doi:10.3390/molecules24071201)
Supplement: Supplementary file 1 [file molecules-24-01201-s001.pdf]

Supporting Information for

Bacteria Death and Osteoblast Metabolic Activity Correlated to Hydrothermally Synthesised TiO2 Surface Properties

Alka Jaggessar[1, 2], Asha Mathew[2], Tuquabo Tesfamichael[1], Hongxia Wang[1], Cheng Yan[1] and Prasad KDV Yarlagadda[1, 2,*]

[1] Science and Engineering Faculty, Queensland University of Technology, 2 George Street, Brisbane QLD 4001, Australia; a.jaggessar@hdr.qut.edu.au (A.J.); t.tesfamichael@qut.edu.au (T.T.); hx.wang@qut.edu.au (H.W.); c2.yan@qut.edu.au (C.Y.)

[2] Institute of Health and Biomedical Innovation, Queensland University of Technology, 60 Musk Avenue, Kelvin Grove QLD 4059, Australia; asha.mathew@qut.edu.au (A.M.)

* Correspondence: y.prasad@qut.edu.au

Table 1: S.aureus average CFU/mL results.

Sample	0 H CFU/mL	3 H CFU/mL	18 H CFI/mL
Control	15014233.99	30609866.88	21228916.92
Flat	15014233.99	25392990.19	20105402.81
1.0_3_240	15014233.99	14055711.46	14511402.5
2.0_3_240	15014233.99	13702158.07	16145604.84
0.1_3_240	15014233.99	37343094.79	494974.7468
1.0_3_120	15014233.99	2223457.99	1241365.238
1.0_1_240	15014233.99	11887250.67	4124789.557
2.0_10_240	15014233.99	15564205.93	8736697.119

Table 2: P.aeruginosa average CFU/mL results.

Sample	0 H CFU/mL	3 H CFU/mL	18 H CFI/mL
Control	329922222.2	25834074074	76752222222
Flat	329922222.2	29689629630	1.06649E+11
1.0_3_240	329922222.2	16922962963	60173333333
2.0_3_240	53216666.67	23402222222	1.29672E+11
0.1_3_240	53216666.67	40871111111	1.23652E+11
1.0_3_120	53216666.67	24856111111	1.22388E+11
1.0_1_240	53216666.67	36184444444	1.29311E+11
2.0_10_240	53216666.67	42376111111	1.41044E+11

Table 3: AlamarBlue™ test results averaged.

Sample	4 H Activity (%)	24 H Activity (%)
Flat	100	100
1.0_3_240	104.429	82.52287
2.0_3_240	104.3596	83.89352
0.1_3_240	111.1907	103.918

1.0_3_120	108.4379	92.82887
1.0_1_240	95.18652	80.75645
2.0_10_240	120.6004	87.88418
